# Personalizing blood pressure management in septic shock

**DOI:** 10.1186/s13613-015-0085-5

**Published:** 2015-11-16

**Authors:** Ryotaro Kato, Michael R. Pinsky

**Affiliations:** Department of Critical Care Medicine, University of Pittsburgh School of Medicine, 606 Scaife Hall, 3550 Terrace Street, Pittsburgh, PA 15261 USA

**Keywords:** Arterial blood pressure, Autoregulation, Critical closing pressure, Organ blood flow, Resuscitation, Sepsis, Septic shock, Vasopressor therapy

## Abstract

This review examines the available evidence for targeting a specific mean arterial pressure (MAP) in sepsis resuscitation. The clinical data suggest that targeting an MAP of 65–70 mmHg in patients with septic shock who do not have chronic hypertension is a reasonable first approximation. Whereas in patients with chronic hypertension, targeting a higher MAP of 80–85 mmHg minimizes renal injury, but it comes with increased risk of arrhythmias. Importantly, MAP alone should not be used as a surrogate of organ perfusion pressure, especially under conditions in which intracranial, intra-abdominal or tissue pressures may be elevated. Organ-specific perfusion pressure targets include 50–70 mmHg for the brain based on trauma brain injury as a surrogate for sepsis, 65 mmHg for renal perfusion and >50 mmHg for hepato-splanchnic flow. Even at the same MAP, organs and regions within organs may have different perfusion pressure and pressure–flow relationships. Thus, once this initial MAP target is achieved, MAP should be titrated up or down based on the measures of organ function and tissue perfusion.

## Background

In 1969, Weil and Shubin emphasized the importance of fluid resuscitation followed by cardiovascular support with vasoactive agents for the treatment of shock [1]. This strategy is still the mainstay of management of septic shock today [2]. The Surviving Sepsis Campaign Guidelines recommend initial resuscitation by fluid administration, at least with 30 ml/kg of crystalloids, followed by use of vasoactive agent such as norepinephrine for the treatment of patients with septic shock [3]. Hypotenison can be defined as a systolic arterial pressure <90 mmHg, a mean arterial pressure (MAP) <65 mmHg or a decrease in MAP >40 mmHg in a previously hypertensive patient [4].

Although this strategy has been well established, blood pressure target in septic shock patients remains a subject of ongoing controversy. The Surviving Sepsis Campaign Guidelines recommend MAP target of 65 mmHg as a starting point [3], but this recommendation is based on limited evidence. The guidelines caution that the MAP target should be individualized because older patients with atherosclerosis or previous hypertension, for example, may have a higher optimal MAP than younger patients without any cardiovascular conditions.

In 2004, Asfar et al. conducted a multicenter, randomized, open-label, prospective study involving 776 septic shock patients in French intensive care units (ICU) [5]. The study confirmed that targeting an MAP of 65–70 mmHg in a patient without prior chronic hypertension was a reasonable first approximation. In a patient with a history of chronic hypertension, however, targeting an MAP of 80–85 mmHg was associated with lower incidences of AKI and the need for renal replacement therapy. Although patients with chronic hypertension benefited from this higher MAP target, it was associated with higher incidences of adverse events such as tachyarrhythmia, presumably because higher doses and duration of vasopressors were necessary.

Taken together, the available evidence underscores the importance of personalizing the MAP target based on clinical responses of individual patients with septic shock. Heterogeneity, not only of patients, but their individual organs and microcirculation [6, 7] makes uniform approach to septic shock particularly difficult. The aim of this review, therefore, is to provide some guidance on how to personalize management of blood pressure in patients with septic shock. We reviewed the existing literatures using both PubMed and Google Scholar search engines for the primary search terms: arterial blood pressure, sepsis, severe sepsis, septic shock, perfusion pressure, critical closing pressure and autoregulation. We then expanded our search as linked citations indicated. We limited these searches to studies on adult patients published in English.

## Review

### Pathophysiology

Humans, like other warm-blooded animals, maintain relatively high blood pressure at the expense of its multiple potentially negative consequences, such as myocardial ischemia, atherosclerosis, aneurysm or chronic kidney disease. This is because high blood pressure is necessary to allow autoregulation of organ blood flow to occur.

Autoregulation is defined as the intrinsic ability of organs to maintain a constant blood flow despite changes in perfusion pressure [8]. Since organs autoregulate their blood flow to meet their metabolic demands, this dissociation between pressure and flow seems reasonable. Organ blood flow and cardiac output (CO) are usually independent of arterial blood pressure except under extreme hypo- and hypertension.

Organs can increase their own individual blood flow to meet their changing metabolic demands primarily by decreasing resistance, or vasodilation. Accordingly, both inflow pressure and intra-organ inflow resistance at the baseline must be sufficiently high to leave sufficient room for autoregulation of organ blood flow to occur. As a corollary, hypotension alone impairs local autoregulation independent of other factors like vasomotor tone and vascular responsiveness because without a sufficiently high inflow pressure, changes in local vascular resistance will not result in changes in local blood flow.

Organ perfusion pressure is the difference between the inflow pressure and outflow pressure. In a totally vasodilated vasculature, outflow pressure approximates local venous pressure. Inflow and outflow pressures differ across vascular beds and can be altered by various diseases (Table 1). Although MAP is usually considered to be the inflow pressure, actual arterial inflow pressure varies greatly across organs. For example, arterial inflow pressure at porta hepatis is about 10–30 mmHg lower than MAP because of high hepatic arterial resistance. Similarly, renal perfusion pressure of the post-glomerular tubules is much lower than MAP and varies greatly based on solute load. Outflow pressure is not uniform across organs either. Global renal perfusion pressure, which is the difference between MAP and central venous pressure (CVP), becomes the difference between MAP and intra-abdominal pressure (IAP) when IAP is elevated, such as in intra-abdominal hypertension or abdominal compartment syndrome.

**Table 1:** Perfusion pressure for different organs

Organs	Inflow pressure	Outflow pressure (whichever is higher)	Perfusion pressure
Brain	MAP	CVP or intracranial pressure (ICP)	MAP—CVP or ICP
Heart	Diastolic BP	CVP or intrathoracic pressure (ITP)	Diastolic BP—CVP or ITP
Kidney	MAP	CVP or intra-abdominal pressure (IAP)	MAP—CVP or IAP
Bowel	MAP	CVP or intra-abdominal pressure (IAP)	MAP—CVP or IAP

*MAP* mean arterial pressure, *BP* blood pressure, *CVP* central venous pressure

Under normal conditions, distribution of organ blood flow is determined by local metabolic demands. For example, cerebral blood flow increases in the cortex when the mind is actively thinking [9], and splanchnic blood flow at the site of peristalsis and absorption increases after a meal [10]. Actively metabolizing tissues are thought to increase blood flow by releasing vasoactive substances such as adenosine, a potent vasodilator [11]. In contrast, under hypotensive conditions, organ blood flow is no longer determined by local metabolic demands, but is redistributed according to each organ’s pressure–flow relationship under maximally vasodilated conditions.

This is because autoregulation, though central for normal blood flow homeostasis, is overruled in circulatory shock where baroreceptor-induced hypotension induces profound sympathetic nervous system output. Thus, in circulatory shock, sympathetic-induced vasoconstriction, not the metabolic-related vasoconstriction, becomes the primary determinant of organ blood flow distribution. The massive sympathetic discharge causes α-adrenergic receptor-based vascular vasoconstriction to occur as a function of the amount of vascular α-adrenergic receptor density and responsiveness of a given vascular region. Skin and skeletal muscle have large concentrations of α-adrenergic receptors and constrict markedly in response to circulatory shock. The gut has less α-adrenergic receptors and the kidneys lesser still. Importantly, the heart has minimal α-adrenergic receptors and the cerebral circulation none. Therefore, in case of severe systemic hypotension, organ blood flow will be diverted away from the skin, non-exercising skeletal muscles and splanchnic viscera to support the brain, heart and kidney blood flow [8]. This redistribution of blood flow not only ensures adequate blood flow to these critical organs, but also increases the net efficiency of O_2_ utilization of a whole body [12]. Importantly, during septic shock, adrenergic hypo-responsiveness often occurs owing to internalization of adrenergic receptors and inflammatory mediator-induced release of potent vasoactive agents (e.g., nitric oxide). The resultant combination of systemic hypotension and vasoplegia blunts the normal redistribution of blood flow usually seen in circulatory shock and markedly limits the host’s ability to sustain the vital organ blood flow. If the perfusion pressure falls below the autoregulation threshold where blood vessels are already maximally dilated, organ blood flow will decrease linearly to declines in perfusion pressure.

**Fig. 1:**
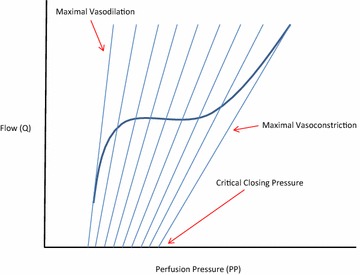
Theoretical relationship between arterial input pressure (*P*) and blood flow (*Q*) for a given vascular bed or the entire body. The *thick solid line* represents the actual relationship between pressure and flow describing the autoregulation of vascular tone to sustain a constant blood flow despite varying arterial input pressures. The *smaller straight lines* reflect the theoretical instantaneous arterial input pressure to blood flow relations that exist upon this autoregulation curve showing how changes in vascular tone from maximal vasoconstriction (*far left*) to maximal vasodilation (*far right*) account for this phenomenon. Note the zero blood flow intercept points, or critical closing pressure of the arterial input circuit also varies with changes in vasomotor tone such that both slope (resistance) and zero-flow intercept (critical closing pressure) co-vary as local vasomotor tone varies

Under normal conditions, if inflow pressure were to be abruptly decreased, organ blood flow would also decrease and then cease at an inflow pressure higher than outflow venous pressure. This organ-specific stop-flow pressure is called critical closing pressure (Pcc) and it was first proposed by Burton [13]. Pcc is generated by vasomotor tone of arterioles and pre-capillary sphincters. As a lump sum, Pcc is thought to be around 45 mmHg in normal healthy adults [14], but it can vary among vascular beds dependent upon the overall sympathetic tone and local metabolic demands. As local vasodilation increases, Pcc decreases toward outflow pressure (Fig. 1). Notably, in the heart, which is maximally extracting oxygen at all times, Pcc is only slightly higher than CVP and the primary way the coronary circulation can increase its flow is by vasodilation [15–17].

**Fig. 2:**
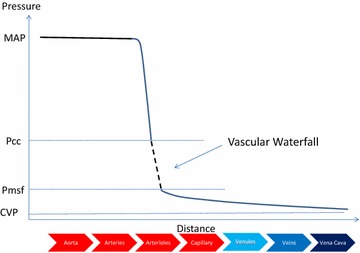
Theoretical vascular pressure profile from aortic values through the circulation to the great veins. Note that mean arterial pressure (MAP) is constant for most of the length of the large arteries, because those vessels serve mainly as vascular capacitors holding stored blood under pressure. Whereas vascular pressure drops rapidly as blood traverses the smallest arteries, arteriole and precapillary sphincters. The point at which arterioles spontaneously collapse limiting arterial pressure drop is referred to as the critical closing pressure (Pcc) and approximates a vascular waterfall, in that water flowing over a waterfall is unaffected by how far it falls once over the edge. Thus, shown as a *dashed line*, the pressure fall from arterioles to venules; changes in the downstream venous pressure do not influence either arterial pressure or blood flow. While the mean systemic filling pressure (Pmsf) represents the upstream pressure driving venous return against a downstream central venous pressure (CVP). These concepts were recently validated in post-operative humans where Pcc was estimated to be about 40 mmHg and Pmsf at 20 mmHg [14]

Under normal resting conditions, perfusion pressure is the difference between inflow pressure and Pcc, and outflow venous pressure does not influence organ blood flow [18]. This phenomenon is called “vascular waterfall.” The principle of vascular waterfall is that flow over the edge of the waterfall is independent on how far the water then drops toward the pool below (Fig. 2). Local tissue Pcc is analogous to the waterfall edge and central venous pressure (CVP) to the downstream pool, such that changes in CVP will have no impact on the flow or resistance. Therefore, while CVP is necessary in calculating organ perfusion pressure, CVP should not guide treatment decisions in patients with septic shock. Maas et al. confirmed the existence of a vascular waterfall by showing a significant difference between Pcc and mean systemic filling pressure (Pmsf) in post-cardiac surgery patients [14]. This difference signified the height of vascular waterfall.

It is critically important to identify patients whose perfusion pressure is below the autoregulation threshold because from the point downward, organ blood flow is usually inadequate and organ perfusion will solely depend on perfusion pressure. This is the rationale for using vasopressors to restore organ perfusion pressure during acute resuscitation in fluid resuscitated patients with septic shock. Regrettably, there is not one threshold MAP because each organ system has a different inflow and outflow pressures and internal control systems linked to their individual physiologic roles [19]. For example, the kidney increases filtration as renal perfusion pressure increases because its role is to filter solute from the blood, whereas the liver maintains a relatively constant flow from the combined hepatic artery and portal vein so as to maintain hepatic clearance and metabolic functions.

### Clinical evidence

Although the Surviving Sepsis Guidelines recommend using vasopressor to support an initial MAP target of 65 mmHg followed by individualized titration [3], this recommendation was based on limited evidence. A retrospective cohort study by Varpula et al. showed that MAP below 65 mmHg, particularly during the first 48 h in the ICU, was associated with the highest mortality in patients with septic shock [20]. Meanwhile, a small prospective study by LeDoux et al. showed no improvements in tissue perfusion by increasing MAP from 65 to 85 mmHg using norepinephrine [21], and a small randomized, open-label, prospective study by Bourgoin et al. also showed lack of any benefit by targeting an MAP higher than 65 mmHg [22].

Looking specifically at renal function, however, other studies found that targeting an MAP higher than 70 mmHg might be beneficial [23, 25]. Furthermore, in reality, the majority of critical care practitioners seemed to be targeting an MAP higher than 65 mmHg [26]. Clearly, more studies were needed to determine the optimal MAP in patients with septic shock.

In this context, Asfar et al. conducted a multicenter, randomized, stratified, open-label study called the Assessment of Two Levels of Arterial Pressure on Survival in Patients with Septic Shock (SEPSISPAM) to determine whether targeting an MAP of 65–70 mmHg was more or less effective than targeting a higher MAP of 80–85 mmHg [5]. Unfortunately, MAP values of the low-MAP target group usually ranged from 70 to 75 mmHg and rarely decreased toward the 65 mmHg minimal threshold. Still, the study showed that there was no significant between-group difference in the rate of death at 28 and at 90 days. For the patients with chronic hypertension, the low-MAP target group had a higher incidence of the doubling of creatinine level and the need for renal replacement therapy.

Importantly, targeting a higher MAP in all patients was not without risk. Although this study was underpowered to detect any differences in incidence of most of the adverse events, which were rare, the majority of adverse events (mainly tachyarrhythmias) were reported higher in the high-MAP target group who required higher infusion rates and duration of vasopressors [5].

The study supports the recommendation that targeting an initial MAP of 65–70 mmHg in a patient without prior chronic hypertension is a reasonable first approximation, after which time MAP levels should be adjusted up or down as end-organ function dictates. Whereas in the patient with chronic hypertension, targeting a higher MAP around 80–85 mmHg appears to be a reasonable first step, but it should be done with caution because of the potential risk of adverse events due to higher doses and duration of vasopressors that would be necessary. Although no study to date has shown the impact of the dose and duration of vasopressors on survival, studies have consistently shown the risk of adverse events due to vasopressor use, ranging from 10 to 12 % [27–29].

These findings underscore the importance of personalizing target MAP based on individual patient’s clinical response. There is no “one-size fits all” when it comes to optimal MAP for septic shock patients. This may seem to be an obvious conclusion, since MAP is not organ perfusion pressure, as described above. In fact, organ perfusion pressure is highly heterogeneous, not only between patients, but also within the same patient over time and among their organs and microcirculation during the evolution of septic shock [6, 7]. This is what makes blood pressure management in septic shock, particularly challenging, requiring close bedside titration.

After the SEPSISPAM study was published, two review articles were published analyzing blood pressure targets for septic shock patients. Leone et al. reviewed 12 studies including 7 comparative studies that addressed different blood pressure goals on patient outcomes [30]. They concluded that MAP target of 65 mmHg is usually sufficient in patients with septic shock, but MAP target of around 75–85 mmHg may reduce the incidence of acute kidney injury (AKI) in patients with chronic hypertension.

D’Aragon et al. also reviewed 12 studies including two randomized control studies, which were the SEPSISPAM study and a Czech study, and 10 crossover studies [31]. They refrained from making any conclusions regarding optimal target blood pressure and commented instead on the paucity of clinical evidence to guide blood pressure management in septic shock patients. They were particularly concerned with prior studies for using limited types of vasopressors, potential inaccuracies on blood pressure measurements and titration of vasopressors based on endpoints other than blood pressure. Their concerns may be justified. Practitioners and researchers tend to disagree even on such a fundamental practice as measuring an MAP [32].

The issue of vasopressor choice needed to support a given target MAP is also relevant to this discussion. It had been suggested that dopamine might increase splanchnic blood flow in well-resuscitated patients with septic shock [33], but SOAP II study failed to show this [29]. In that study, they compared dopamine to norepinephrine in the management of vasopressor-dependent septic shock. Although they showed no mortality difference between the two study arms, the group receiving dopamine had a higher rate of arrhythmias and many patients in that group also required supplemental norepinephrine to reach their target MAP goals. Based on these data, the authors and the Surviving Sepsis Guidelines both recommend norepinephrine as the vasopressor of choice. Likewise, it had been suggested that vasopressin might impair hepato-splanchnic blood flow [34]. As such, VASST trial studied the addition of vasopressin to usual vasopressor management. They showed no differences in the rate of hepatic dysfunction or mesenteric ischemia [28]. A smaller prospective randomized study even showed better splanchnic perfusion with vasopressin as compared to norepinephrine alone [35]. Currently, another trial is underway, comparing vasopressin with or without corticosteroids to norepinephrine as the initial vasopressor in the management of patients with septic shock [36].

Meanwhile, it has been suggested that vasodilators such as prostacyclin may improve hepato-splanchnic circulation [35]. In a small prospective study involving septic shock patients requiring norepinephrine to maintain MAP above 70 mmHg, prostacyclin (PGI_2_ or iloprost) infusion showed improvement in both cardiac output and hepato-splanchnic blood flow [37]. Notably, the actual median MAP was around 80 mmHg in this study.

### Organ-specific blood flow considerations

#### Brain

As early as the time of Hippocrates more than 2500 years ago, sepsis has been known to affect brain function [38]. Sepsis-associated delirium is the most common brain dysfunction and it can be found in up to 70 % of septic patients [38, 39]. It is associated with a significant increase in mortality [40, 41].

**Fig. 3:**
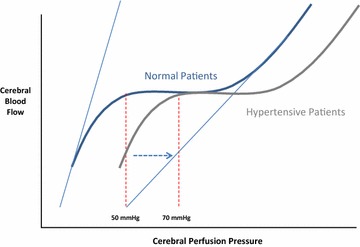
Theoretical relationship between cerebral perfusion pressure (CPP) and cerebral blood flow using the same construct as in Fig. 1. Here, the autoregulatory range for subjects without hypertension (normal patients) is in blue and that for patients with hypertension (hypertensive patients) is shown in gray. Note that the minimal CPP within the autoregulatory zone for normal is about 50 mmHg whereas for those with hypertension it is shifted rightward with CPP on the *x-axis* to 70 mmHg. Again the maximal vasoconstriction and vasodilation instantaneous CCP-cerebral blood flow relations for normal patients are shown as the *light blue lines*

Cerebral perfusion pressure (CPP) is defined as the difference between MAP and either CVP or intracranial pressure (ICP), whichever is higher. Under normal conditions, the brain maintains a high degree of autoregulation [8]. Notably, in patients with preexisting cerebrovascular conditions such as chronic hypertension, the autoregulation threshold is shifted significantly to the right by as much as 20 mmHg (Fig. 3) [42].

A study using transcranial Doppler and near-infrared spectroscopy showed that cerebral autoregulation is disturbed in severe sepsis, presumably due to vascular endothelial dysfunction [43]. Cerebral blood flow is also reduced in severe sepsis [44]. Although its precise mechanism is yet to be understood, Pfister et al. demonstrated that sepsis-induced cerebral edema can increase ICP to more than 15 mmHg, resulting in CPP less than 60 mmHg [45].

There are no clinical studies looking specifically at optimal MAP for the brain in severe sepsis, but a growing body of evidence looking at the relationship between CPP and outcomes in patients with traumatic brain injury (TBI) may prove helpful. Based on multiple indices such as brain tissue O_2_ saturation, jugular venous oxygen saturation, transcranial Doppler and cerebral microdialysis studies, autoregulation threshold for CPP is thought to be around 50–60 mmHg [46, 47].

Accordingly, Brain Trauma Foundation recommends a target CPP between 50 and 70 mmHg [48]. Within this range, however, results of the existing studies are conflicting. For example, one retrospective study involving 392 patients with severe brain injury showed the poor outcome associated with CPP below 60 mmHg [49], while another retrospective study involving 427 patients with severe head injury showed no benefit in keeping CPP above 60 mmHg [50].

One exciting development in the management of CPP in TBI patients is the emergence of autoregulation-based therapy using cerebrovascular reactivity, which can be determined by looking at response of ICP to changes in MAP [51]. Loss of cerebrovascular reactivity is an independent predictor of fatal outcome following head injury [52]. Using real-time measurements of pressure reactivity index, Steiner et al. found a target CPP in head injury patients to be between 60 and 85 mmHg [51]. The autoregulation range of an individual patient is much narrower, however [53]. Accordingly, the importance of titrating a target CPP based on pressure vascular reactivity index in individual TBI patients was suggested [54]. A similar approach that targets autoregulation rather than an MAP may also be useful in septic shock patients.

#### Heart

Sepsis-induced myocardial depression is common and it tends to appear later in the course of the disease. Initially, patients with severe sepsis present with reduced CO, despite the preserved left ventricular ejection fraction because stroke volume is reduced as a result of decreased preload and vasomotor tone [55, 56]. Both these processes cause venous return to the heart to markedly decrease. Volume resuscitation is critical in these patients and usually restores stroke volume to baseline and CO to baseline or even higher levels owing to a combined tachycardia and peripheral vasodilation.

Later in their course, typically within the first 72 h, 40–50 % of these patients develop myocardial depression [57]. Their CO, however, is often increased because the reduction in left ventricular ejection fraction is compensated by tachycardia and dilated ventricles [58]. Sepsis-induced myocardial depression is reversible and full recovery of cardiac function is typically seen in survivors by 7–10 days [59].

Pathophysiology of sepsis-induced myocardial depression is complicated and involves various mechanisms, such as downregulation of β-adrenergic receptors, decreased sensitivity to calcium or increased nitric oxide production [58, 60–62]. Notably, ischemia is not one of the etiologies listed. Coronary blood flow is increased in severe sepsis and myocardial oxygen consumption appears to be adequate [56, 63, 64]. Neither cellular hypoxia nor bioenergetics failure has been seen in a septic heart [65].

These findings led to a hypothesis that perhaps sepsis-induced myocardial depression is an adaptive response by which human heart attempt to prevent activation of cell death pathways and to allow full functional recovery by reducing energy expenditure [66].

If that would be the case, the best management of heart in sepsis may be to avoid further stresses on the heart. Interestingly, recent randomized control trial showed improved clinical outcome using β-blockers in septic shock patients [67]. While its exact mechanism remains unknown, the trial showed that β-blockade could make the heart more efficient, as evidenced by improved stroke work index and left ventricular stroke work [68]. This is an area of active clinical study.

#### Kidneys

Renal function may be the most studied with regard to target blood pressure in patients with severe sepsis or septic shock. Early animal study by Robertson et al. had shown that autoregulation threshold of kidneys might be around 80 mmHg [69]. At least two subsequent human studies seemed to confirm this by showing improved creatinine clearance in septic shock patients whose baseline MAP below 60 mmHg was raised above 80 mmHg using norepinephrine [70, 71]. Looking specifically at urine output, however, LeDoux et al. showed that raising MAP from baseline 65 mmHg to 85 mmHg using norepinephrine conferred no benefit [21]. This finding was confirmed by a small prospective randomized control study by Bourgoin et al. involving 28 patients [22]. These clinical studies implied that autoregulation threshold of kidneys may be closer to 65 mmHg than 80 mmHg.

More recent studies seemed to favor somewhere in the middle. A larger, but retrospective clinical study by Dünser et al. showed that MAP below 75 mmHg was associated with a higher requirement of renal replacement therapy [23]. Notably, 38 % of the study population had chronic arterial hypertension. Badin et al. also showed in their prospective cohort study that optimal MAP to prevent AKI was somewhere between 72 mmHg and 82 mmHg [24]. Prevalence of chronic hypertension in their study was not reported. Another large prospective observational study by Poukkanen et al. suggested that MAP below 73 mmHg was associated with progression of AKI [25]. Nearly half of their study population had chronic hypertension and the overall rate of AKI was high at 36.2 %.

In elderly patients or patients with hypertension, atherosclerosis or chronic kidney diseases, the autoregulation curve of kidneys can be shifted significantly to the right [72, 73]. This may be why the above studies showed benefit of higher MAP to prevent AKI when the study cohort included many patients with chronic hypertension.

To confirm this, Asfar et al. stratified their study population at the time of randomization according to whether or not they had a history of chronic hypertension [5]. More than 40 % of the study population had chronic hypertension. Indeed, they found that among the patients with chronic hypertension, the low-MAP target group had a significantly higher rate of doubling of creatinine or need for renal replacement therapy, compared to the high-MAP target group.

Notably, what is missing in all of these studies is the consideration of IAP. The renal perfusion pressure becomes the difference between MAP and IAP when IAP exceeds CVP. IAP can be measured at the bedside using bladder pressure [74, 75]. IAH is defined as a sustained elevation of IAP above 12 mmHg whereas normal IAP is considered to be approximately 5–7 mmHg [76]. Sustained IAP above 20 mmHg is called abdominal compartment syndrome (ACS) and results in intra-abdominal organ dysfunction [76]. Thus, in case of ACS, such as in abdominal sepsis or sepsis associated with liver failure, MAP target may need to be increased at least by the increase in IAP.

Given the current state of evidence, MAP target of 65 mmHg may be reasonable in septic shock patients without any heightened susceptibility to AKI, such as preexisting chronic hypertension, atherosclerosis, chronic kidney disease or advanced age. In contrast, septic shock patients with these risk factors may benefit from higher target MAP of 80 mmHg. In patients with IAH, further increase in MAP target may be necessary, depending on their IAP. Notably, multiple other conditions including the use of medications such as nonsteroidal anti-inflammatory drugs can also impair kidney’s autoregulation and may benefit from higher MAP.

Vasopressor of choice to achieve desired MAP is norepinephrine. Norepinephrine reduces renal blood flow in normal condition, but increases in sepsis by both decreasing renal vascular resistance and Pcc [77]. Vasopressor should be used with caution, however, because of its potential complications as mentioned above. This point may be particularly important because an increasing body of evidence suggests that hypotension, though important, may not be the primary cause of sepsis-associated AKI [78]. Schlichtig et al. showed that kidneys could tolerate significant hypotension compared to rest of the body [79]. In a large retrospective cohort study, Murugan et al. found that sepsis-associated AKI can occur in the absence of global hypotension [80]. Restoration of hemodynamic variables alone thus should not be the only goal for the management of sepsis-associated AKI [81].

#### Liver

The liver plays a critical role in severe sepsis and septic shock for two reasons [82]. First, the entire splanchnic circulation, which comprises 25 % of cardiac output, must pass through the liver. This is particularly important because the gastrointestinal tract is thought to be the driver of multi-organ failure syndrome in sepsis [83]. Second, nearly 90 % of the body’s reticuloendothelial system exists within the liver, primarily as Kupffer cells [82]. Thus, the liver is thought to be the clearinghouse of microbial pathogen-associated molecular patterns (PAMPs) and endogenous “damage”-associated molecular patterns (DAMPs) that incite and perpetuate systemic inflammatory response [84].

Unlike the other organs, but like the lungs, the liver receives both arterial and venous blood flow. Hepatic artery supplies 25–50 % of hepatic blood flow and portal vein supplies the remainder [85]. Regulation of hepatic arterial flow and portal venous flow is distinct from each other. Autoregulation is the primary mechanism for the arterial system, while distensibility of vascular beds that create capacitance and existence of vascular waterfall in the portal venous system allows steady venous return despite changes in CVP [86, 87]. Furthermore, any reduction in portal venous flow is mitigated by a reciprocal increase in hepatic arterial blood flow. This mechanism is called hepatic arterial buffer response and appears to be mediated by adenosine [88].

Severe sepsis affects the liver in two stages [89]. In the first hours, early hepatic dysfunction occurs due to hypoperfusion. Both autoregulation of hepatic artery and hepatic buffer response appear to be impaired [90]. This is followed by late hepatic dysfunction, characterized by functional and structural injury due to various circulating PAMPs and DAMPs.

Often, hepatic and splanchnic circulations are studied together due to technical difficulties in isolating one from another [33], and studies specifically looking at optimal MAP for hepato-splanchnic circulation in septic shock are limited. In Asfar et al., there was no difference in the rate of mesenteric ischemia between the low-target group and the high-target group (2.3 versus 2.3 %) [4]. A study that showed the lowest rate of bowel ischemia was the SOAP II study, which compared dopamine versus norepinephrine for the treatment of septic shock (1.3 versus 0.7 %) [29]. Notably, the actual MAP was maintained only around 58 mmHg in this study. In other studies that compared various regimens of vasopressors and their impact on hepato-splanchnic circulation, the actual MAP was maintained at least above 70 mmHg [33].

## Conclusion

The available evidence suggests that targeting an MAP of 65–70 mmHg in a patient with septic shock who does not have chronic hypertension is a reasonable first approximation. Whereas in a patient with chronic hypertension, targeting an MAP of 80–85 mmHg appears to be a reasonable first step. It must be done with caution, however, because the use of vasopressors is associated with adverse events. After these initial treatments, MAP should be titrated up or down based on the individual patient’s response, but heterogeneity, not only of patients, but of organs and microcirculations affected by septic shock makes it challenging. Caution needs to be taken in all patients in using MAP alone as surrogate of organ perfusion pressure, especially under conditions in which intracranial or intra-abdominal pressure may be elevated.

If sepsis-associated delirium is the primary concern, a growing body of evidence regarding the management of traumatic brain injury patients suggests that MAP target between 50 and 70 mmHg is needed. Further titration of MAP based on multi-modal monitoring including pressure-reactivity index may worth further investigation.

Sepsis-induced myocardial depression, though challenging in its management, may be an adaptive response by the heart in sepsis. Because coronary blood flow is increased, MAP target should not guide its management. Rather, it is important not to stress the heart any further by avoiding excessive or prolonged use of vasopressors. Recent study suggests promising role of β-blockers.

Target MAP in septic shock is most studied in relation to sepsis-associated AKI. In patients without chronic hypertension, atherosclerosis, chronic kidney disease or advanced age, MAP target of 65 mmHg may be reasonable. For patients with these risk factors, MAP target of 80 mmHg may be better. It should be noted, however, that recent evidence suggests that hypotension may not be the primary mechanism of sepsis-associated AKI.

Finally, hepatic dysfunction or mesenteric ischemia that can be associated with septic shock may not only result from the disease, but also from excessive use of vasopressors. MAP target as low as 60 mmHg may be reasonable to reduce vasopressor requirement. Furthermore, there may be a role of vasodilator in the management of hepato-splanchnic blood flow.

## Abbreviations

ACS: abdominal compartment syndrome
AKI: acute kidney injury
CO: cardiac output
CPP: cerebral perfusion pressure
CVP: central venous pressure
DAMPs: damage-associated molecular patterns
IAH: intra-abdominal hypertension
IAP: intra-abdominal pressure
ICP: intracranial pressure
ICU: intensive care unit
MAP: mean arterial pressure
PAMPs: pathogen-associated molecular patterns
Pcc: critical closing pressure
Pmsf: mean systemic filling pressure
TBI: traumatic brain injury
